# Toll-Like Receptor (TLR) 2 and TLR4 Differentially Regulate Doxorubicin Induced Cardiomyopathy in Mice

**DOI:** 10.1371/journal.pone.0040763

**Published:** 2012-07-13

**Authors:** Yonggang Ma, Xiaowei Zhang, Huayan Bao, Su Mi, Wenfeng Cai, Huimin Yan, Qingqing Wang, Ziyan Wang, Jun Yan, Guochang Fan, Merry L. Lindsey, Zhuowei Hu

**Affiliations:** 1 Molecular Immunology and Pharmacology Group, State Key Laboratory of Bioactive Substances and Functions of Natural Medicines, Institute of Materia Medica, Chinese Academy of Medical Sciences & Peking Union Medical College, Beijing, People’s Republic of China; 2 San Antonio Cardiovascular Proteomics Center, Barshop Institute of Longevity and Aging Studies, and Division of Geriatrics, Gerontology and Palliative Medicine, Department of Medicine, The University of Texas Health Science Center at San Antonio, San Antonio, Texas, United States of America; 3 Department of Pharmacology and Cell Biophysics, University of Cincinnati College of Medicine, Cincinnati, Ohio, United States of America; University of Western Ontario, Canada

## Abstract

Recent evidence indicates that toll-like receptor (TLR) 2 and 4 are involved in the pathogenesis of dilated cardiomyopathy (DCM), but the exact mechanisms of their actions have not been elucidated. We explored the therapeutic potential of blocking TLRs in mice with established cardiomyopathy. Cardiomyopathy was generated by a single intraperitoneal injection of doxorubicin (10 mg/kg). Two weeks later, the mice were treated with TLR2 or TLR4 neutralizing antibody. Blocking TLR2, but not TLR4, activity not only reduced mortality, but also attenuated doxorubicin-induced cardiac dysfunction by 20% and inhibited myocardial fibrosis. To determine the differential effects of blocking TLR2 and TLR4 in chronic cardiomyopathy, mice were injected with doxorubicin (3.5 mg/kg) once a week for 8 weeks, followed by treatment with TLR2 or TLR4 neutralizing antibody for 40 days. Blocking TLR2 activity blunted cardiac dysfunction by 13% and inhibited cardiac fibrosis, which was associated with a significant suppression of myocardial inflammation. The underlying mechanism involved interrupting the interaction of TLR2 with its endogenous ligands, resulting in attenuation of inflammation and fibrosis. In contrast, blocking TLR4 exacerbated cardiac dysfunction and fibrosis by amplifying inflammation and suppressing autophagy. Our studies demonstrate that TLR2 and TLR4 play distinct roles in the progression of doxorubicin-induced DCM. TLR4 activity is crucial for the resolution of inflammation and cardiac fibrosis, while blocking TLR2 activity has therapeutic potential for the treatment of DCM.

## Introduction

Dilated cardiomyopathy (DCM), a common cause of chronic heart failure (CHF), is characterized by progressive myocardial remodeling and a decline in cardiac function [1]. Currently, the five year mortality rate for patients with DCM is approximately 50%. While numerous studies have documented that DCM has idiopathic and genetic origins, cardiac inflammation also induces DCM [2]. Myocardial inflammation can be caused by viral and bacterial infection as well as non-infectious factors such as lupus or other autoimmune diseases [3].

Multiple toll-like receptors (TLRs) are expressed in cardiomyocytes, including TLRs 2 and 4. Through these TLRs, cardiomyocytes respond to endogenous or exogenous signals which may influence the pathophysiological responses to DCM [4], [5]. The expression or activation of both TLR2 and TLR4 are up-regulated in experimental models with hypertension and clinical patients with heart failure [6], [7]. Hence, inhibition of TLR signaling may be of great therapeutic benefit for CHF.

Doxorubicin (Dox) is an effective anti-tumor agent. Despite its use as a common chemotherapeutic agent, Dox use can also lead to cardiotoxicity. Multiple intravenous Dox treatments over a period of several months have been shown to induce cardiomyopathy and CHF in humans [8]. Our laboratory and others have also shown that mice and rats treated with 10–30 mg/kg Dox developed DCM [9], [10].

**Figure 1:**
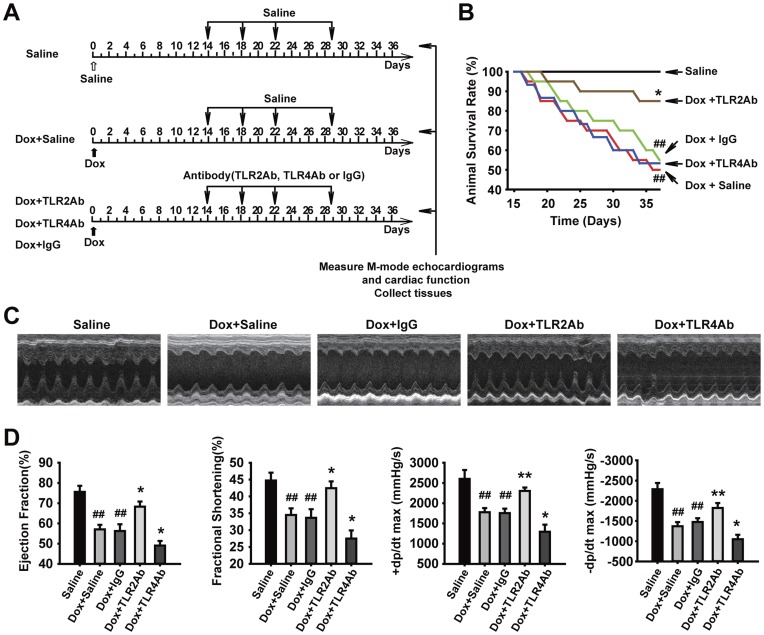
Therapeutic blockade of TLR2 and TLR4 caused opposite effects on cardiac dysfunction and survival in acute DCM. (A) Flowchart for acute DCM and corresponding treatments. (B) Survival rate curves were created by Kaplan-Meier method and compared by log-lank test (n = 15/group). (C) Representative images of LV M-mode echocardiograms. (D) Cardiac function parameters measured by echocardiography and hemodynamic analysis. EF = ejection fraction; FS = fractional shortening; +dp/dtmax = the maximum ascending rate of LV pressure; -dp/dtmax = the maximum descending rate of LV pressure. Data are shown as mean ± SEM (n = 8/group). ##p<0.01 versus saline-treated group; *p<0.05, **p<0.01 versus Dox+IgG-treated group.

Although the precise mechanisms underlying Dox cardiotoxicity have not been fully elucidated, it is widely accepted that Dox-induced cardiac injury is mediated by reactive oxygen species [11], which could be generated from Dox activation of TLR2 or TLR4 signaling pathways [12], [13]. The TLR-mediated innate early inflammatory response is engaged during various forms of heart failure. For example, stimulation of TLR4 by circulating lipopolysaccharide during sepsis reduces ejection fraction [14]. Activation of TLR2 by ischemia/reperfusion leads to post-ischemia dysfunction of the left ventricle (LV) [15]. TLR2 stimulation causes cardiomyocyte singlet oxygen generation and nuclear factor kappa B activation with the expected pro-inflammatory consequences [16]. Accordingly, ablation of TLR2 or TLR4 attenuates ischemia and reperfusion-, LPS-, and Dox-induced myocardial damage [12], [13], [17]. While these findings demonstrate that TLR deficiency is feasible and effective for the prevention of cardiac dysfunction, the therapeutic potential of targeting TLRs in heart failure remains to be defined.

**Figure 2:**
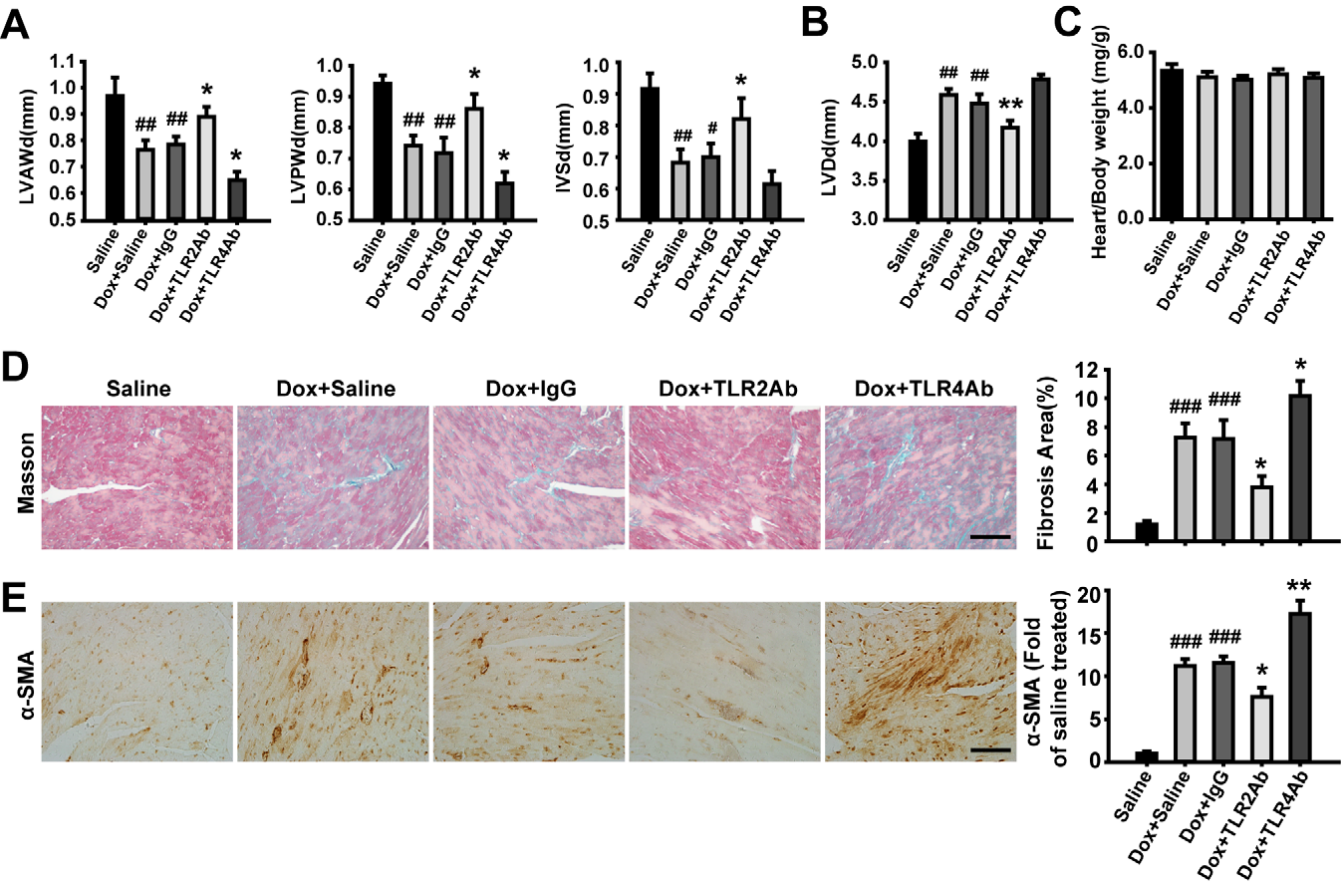
Therapeutic blockade of TLR2 and TLR4 showed opposite effects on cardiac remodeling in acute DCM. (A, B) Wall thickness and LV dimensions measured by echocardiography. LVAWd = left ventricular anterior wall in diastole; LVPWd = left ventricular posterior wall in diastole; IVSd = interventricular septum in diastole; LVDd = left ventricular diameter in diastole. (C) The ratio of heart weight to body weight (mg/g). (D) Masson’s Trichrome staining for cardiac fibrosis evaluation. Scale bar = 50 µm. (E) Expression of α-SMA assessed by immunohistochemistry. Scale bar = 50 µm. Data are mean ± SEM (n = 8–10/group). #p<0.05, ##p<0.01, ###p<0.001 versus saline-treated group; *p<0.05, **p<0.01 versus Dox+IgG-treated group.

In this study, we hypothesized that blockade of TLR2 or TLR4 would have therapeutic benefits against Dox-induced cardiac dysfunction and remodeling. Surprisingly, our findings demonstrated that targeting TLR2 and TLR4 revealed distinct consequences on Dox-induced myocardial injury by differentially regulating inflammatory and autophagic responses.

## Materials and Methods

### Ethics Statement

The animal protocol was approved by the Institutional Animal Care and Use Committee at Peking Union Medical College and conforms to the Guide for the Care and Use of Laboratory Animals published by the US National Institutes of Health.

**Figure 3:**
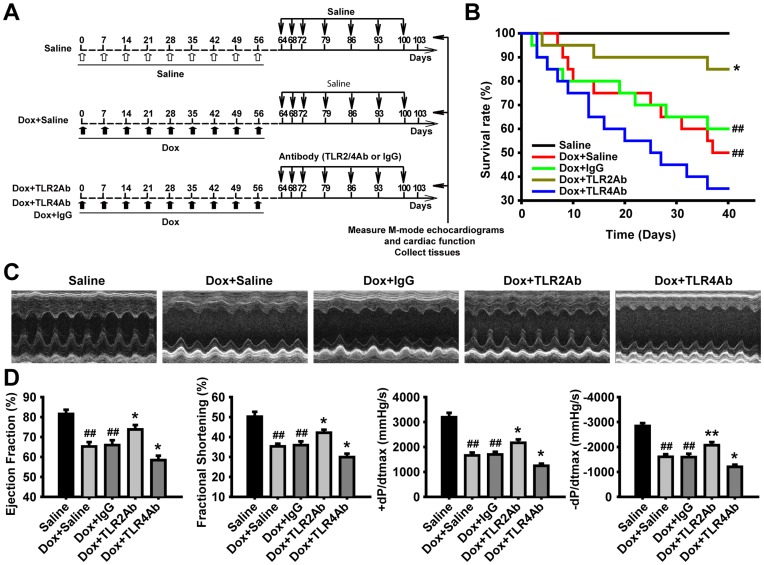
Therapeutic blockade of TLR2 and TLR4 caused opposite effects on cardiac function and survival in chronic DCM. (A) Flowchart for chronic DCM and corresponding treatments. (B) Survival rate curves were created by Kaplan-Meier method and compared by log-lank test (n = 20/group). (C) Representative images of LV M-mode echocardiograms. (D) Cardiac function parameters measured by echocardiography and hemodynamic analysis. Data are mean ± SEM (n = 8/group). ##p<0.01 versus saline-treated group; *p<0.05, **p<0.01 versus Dox+IgG-treated group.

**Figure 4:**
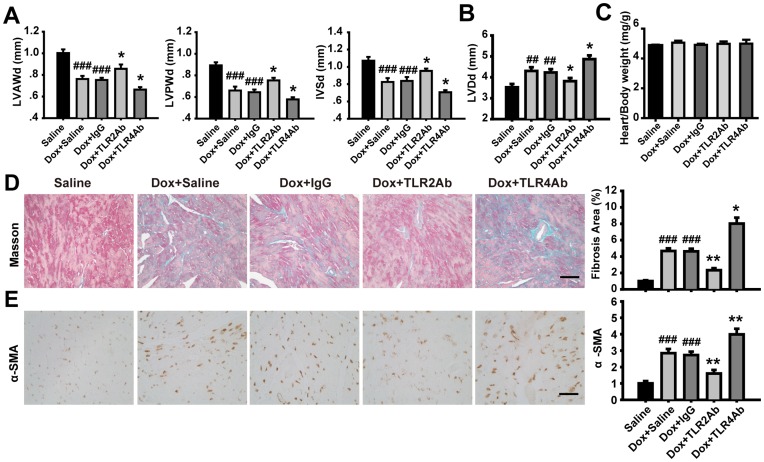
Therapeutic blockade of TLR2 and TLR4 showed opposite effects on cardiac remodeling in chronic DCM. (A, B) Wall thickness and LV dimensions measured by echocardiography. (C) The ratio of heart weight to body weight (mg/g). (D) Masson’s Trichrome staining for cardiac fibrosis evaluation. Scale bar = 50 µm. (E) Expression of α-SMA assessed by immunohistochemistry. Scale bar = 50 µm. Data are mean ± SEM (n = 8/group). ##p<0.01, ###p<0.001 versus saline-treated group; *p<0.05, **p<0.01 versus Dox+IgG-treated group.

### Animal Model and Treatments

Male ICR and C57BL/6J mice were obtained from Vital River (Beijing, China). All mice were housed in a facility with a 12-hour/12-hour light/dark cycle and given free access to water and standard rodent chow. The room was kept specific pathogen-free. Our pilot studies showed that C57BL/6J mice were more susceptible and variable to large doses of Dox than ICR in acute cardiomyopathy induced by doxorubicin. Therefore, we used ICR mice for the acute cardiomyopathy studies. C57BL/6J mice were used for the chronic cardiomyopathy studies.

**Figure 5:**
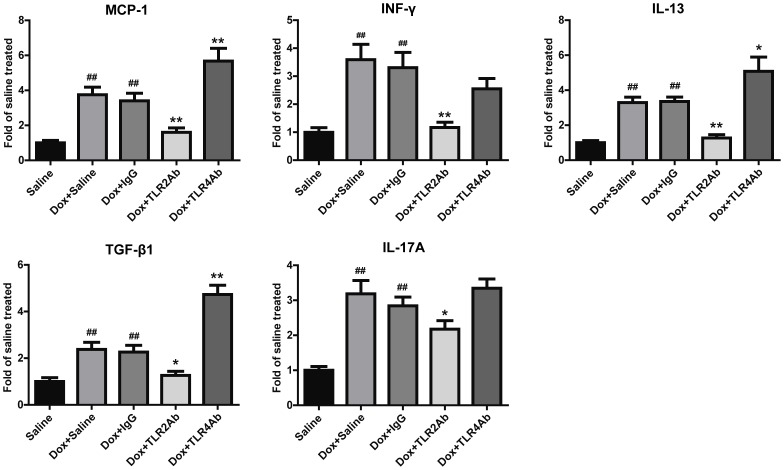
Blockage of TLR2 and TLR4 showed differential regulation on cardiac inflammation in chronic DCM. Data are shown as mean ± SEM (n = 8/group). ##p<0.01 versus saline-treated group; *p<0.05, **p<0.01 versus Dox+IgG-treated group.

Acute cardiomyopathy was induced in 10-week old ICR male mice by a single intraperitoneal (i.p.) injection of Dox at a dose of 10 mg/kg. The mice were treated with TLR2 (R&D, MAB1530), TLR4 (Biolegend, 117608), isotype-matched IgG antibody (SouthernBiotech, 0108-01), or saline on days 14, 18, 22 and 29 after Dox injection. The dosing and timing of antibodies (Abs) were determined based on our previous studies [18], [19]. The antibodies were dissolved in sterile saline, and administered by tail vein injection at days 14, 18, 22 and 29. The first dose was 200 µg/kg, and the remaining doses were 100 µg/kg. At day 36 after Dox injection, LV structural and functional changes were measured by echocardiography and hemodynamic evaluations. The mice were euthanized by using an overdose of pentobarbital (i.p., 100 mg/kg) injection, and the hearts were excised and sliced into several sections for the indicated analyses as described below.

**Figure 6:**
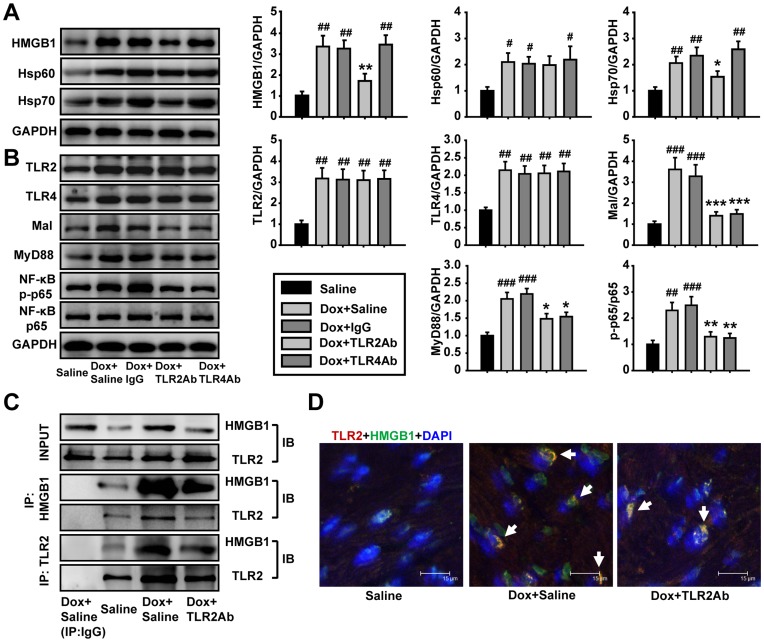
TLR2Ab suppressed the expression of endogenous TLR2 agonists and interaction of TLR2 with HMGB1. (A) Expression of HMGB1 and Hsp70 were reduced in TLR2Ab-treated mice, but not in TLR4Ab group. (B) TLR2Ab and TLR4Ab suppressed its downstream signaling pathway. Data are shown as mean ± SEM (n = 5/group). (C) TLR2Ab inhibited the interaction of TLR2 with HMGB1 as indicated by co-immunoprecipitation and western blot. Data are representatives of two experiments with identical results. (D) TLR2Ab decreased the co-localization of TLR2 and HMGB1 as shown by confocal microscopy. Data are representatives of two experiments with identical results. #p<0.05, ##p<0.01, ###p<0.001 versus saline-treated group; *p<0.05, **p<0.01, ***p<0.001 versus Dox+IgG-treated group.

Chronic cardiomyopathy was induced in 8-week old C57BL/6J male mice by i.p. injection of Dox (3.5 mg/kg) weekly for 8 weeks [20], whereas control mice received equal volume of saline. At day 63 after the initial Dox injection, the mice were randomly divided into 4 groups and treated with TLR2, TLR4, isotype-matched IgG Ab or saline for additional 40 days. The antibodies were dissolved in sterile saline, and administered by tail vein injection at days 64, 68, 72, 79, 86, 93 and 100. The first dose was 200 µg/kg and following doses were 100 µg/kg. At the experimental endpoint, LV structural and functional changes were evaluated by echocardiography and hemodynamic measurements. The mice were euthanized by using an overdose of pentobarbital (i.p., 100 mg/kg) injection, followed by the hearts were excised and sliced into several sections for further analysis.

**Figure 7:**
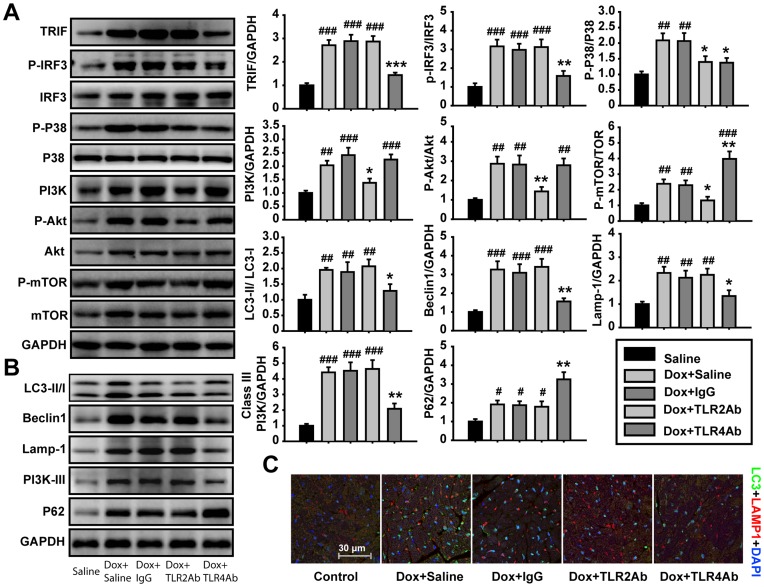
Blockade of TLR2 and TLR4 differentially regulated autophagic activity. (A) Differential roles of TLR2 or TLR4 inhibition on signaling pathways (n = 5/group). (B) Therapeutic blocking TLR4 but not TLR2 activity inhibited autophagy in the Dox-induced DCM hearts (n = 5/group). (C) Co-localization of LC3 and lamp-1 was measured by confocal microscopy. Data are representatives of two experiments with consistent results. Data are shown as mean ± SEM. #p<0.05, ##p<0.01, ###p<0.001 versus saline-treated group; *p<0.05, **p<0.01, ***p<0.001 versus Dox+IgG-treated group.

### Echocardiographic and Hemodynamic Measurements

At the end of the experiment, mice were anesthetized with sodium pentobarbital (i.p., 45 mg/kg), and transthoracic echocardiography was performed using a Vevo 770 High Resolution Imaging system (VisualSonics) with a 30-MHz image transducer. All images were acquired at a heart rate of at least 400 bpm to acquire measurements under a physiologically relevant condition. Measurements were taken from parasternal short axis M mode recordings of the LV. After echocardiography, a PE-10 catheter (Becton Dickinson, USA) was inserted into the LV through the right common carotid artery for continuous recording of LV pressure. Hemodynamic measurements were recorded and analyzed using MPA2000 physiological signal acquiring system (Alcolt Biotech). The maximum ascending and descending rate of LV pressure (±dP/dtmax) were measured as the representative indices of LV systolic and diastolic function. The mice were euthanized by using an overdose of pentobarbital (i.p., 100 mg/kg) injection, followed by the hearts were excised and sliced into several sections for further analysis.

**Figure 8:**
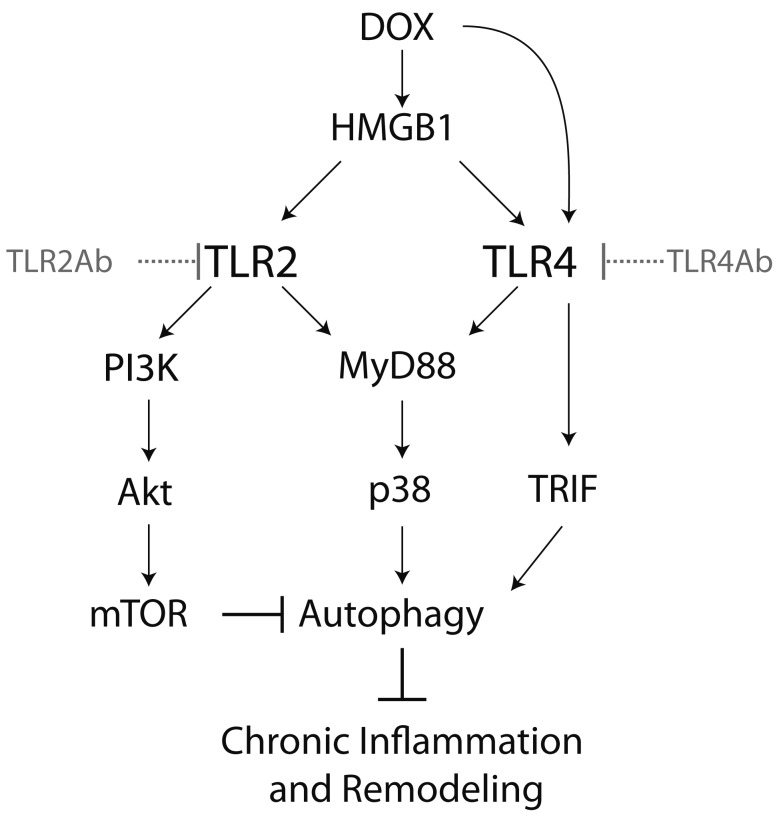
The schematic illustrates mechanism for the differential efficacy of TLR2 and TLR4 antagonism on DCM. Inactivation of TLR4 blocks both MyD88-p38 kinase and TRIF-IRF3 pathways, which results in the suppression of autophagy. In contrast, inactivation of TLR2 induces a biphasic regulation on autophagic activity, due to the inhibition of MyD88-p38 kinase and PI3K-AKT-mTOR pathways.

### Morphological and Histological Evaluation

The tissue was harvested and fixed with 4% paraformaldehyde, embedded in paraffin. Sections (4 µm) were stained by hematoxylin-eosin (H&E), Masson’s trichrome for evaluation of routine morphology and fibrosis. Collagen fractions were calculated as the ratio of collagen area to total ventricular area in the corresponding section; ten random regions per sample at a magnification of 200 × were chosen to determine the average percentage of collagen area (n = 8–10/group, 10 regions/sample). Paraffin-embedded sections were deparaffinized in xylene and rehydrated through graded ethanol. Heat mediated antigen retrieval was performed to expose antigen epitopes. Sections were incubated with a primary antibody against α-smooth muscle actin (SMA, Abcam, ab5694), monocyte chemotactic protein (MCP)-1 (Abcam, ab7202), interferon (IFN)-γ (Abcam, ab24979), interleukin (IL)-13 (Abcam, ab106732), transforming growth factor (TGF)-β1 (Santa Cruz, sc-31609) or IL-17A (Biolegend, 506901). An ABC kit was used for amplification and 3, 3-diaminobenzidine (DAB) was used for staining. Isotype-matched IgG were used as negative controls. The sections were scanned at 200 × magnification. The images were then digitalized and the intensity of densitometry (IOD) were quantified by Image-Pro Plus (Media Cybernetics) [7]. The quantitative analysis was performed by an experienced researcher who was blinded to the different treatment groups.

### Immunoblot

Total protein was extracted by homogenizing samples in 1× RIPA buffer (CST, #9806) with 1 mM phenylmethylsulfonyl fluoride (PMSF) and protease inhibitor cocktail. Protein concentrations were determined with the Coomassie Plus reagent. Total proteins (40 µg) were separated by SDS-PAGE, transferred to PVDF membranes. The membranes were then incubated overnight with primary antibodies against TLR2 (R&D, MAB1530), TLR4 (Biolegend, 117608), MyD88 adapter like (Mal, Santa Cruz, sc-28822), MyD88 (Santa Cruz, sc-11356), p-p65 NF-κB (CST, #3033), p65 NF-κB (CST, #3034), high mobility group box (HMGB)1 (Sigma, H9664), heat shock protein (Hsp) 60 (Abcam, ab13532), Hsp70 (Stressgen, C92F3A-5), LC3 (Abcam, ab51520), beclin1 (CST, #3738), lamp-1 (Abcam, ab25245), PI3K-III (CST, #3811), p62 (Santa Cruz, sc-25575), TRIF (CST, #4596), P-IRF3 (CST, #4947), IRF3 (CST, #4962), P-P38 (CST, #9215), P38 (CST, #9212), PI3K (Santa Cruz, sc-423), P-Akt (CST, #9275), Akt (CST, #9272), P-mTOR (CST, #2971), mTOR (CST, #2972) or GAPDH (Abcam, ab9485). Then, the membranes were incubated with HRP-conjugated secondary antibody, followed by the detection of signal with an enhanced chemiluminescence detection system (Amersham Biosciences).

### Co-immunoprecipitation

For the co-immunoprecipitation experiments, protein lysates extracted from heart homogenates with 1× RIPA buffer supplemented with 1 mM PMSF and protease inhibitor cocktail, were centrifuged for 30 min at 12000 rpm at 4°C. The HMGB1 or TLR2 antibody (2 µg) was added to 700 µl diluted lysates, incubated 2 hours on a rotary wheel at 4°C. Protein A/G PLUS-Agarose beads (Santa Cruz, sc-2003) were added and incubated overnight on a rotary wheel at 4°C. The agarose beads were washed 5 times with the 1× RIPA buffer, solubilized in 2× SDS sample buffer, denatured at 95°C for 5 min, processed for immunoblotting [21].

### Confocal Microscopy

Acetone fixed frozen slides (5 µm) were balanced in PBS for 15 min at room temperature, followed by blocking with 3% BSA. The sections were then incubated with rabbit anti-mouse HMGB1 and goat anti-mouse TLR2 antibody (Santa Cruz, sc-10739), or rabbit anti-mouse LC3 and rat anti-mouse lamp-1 overnight at 4°C. Specific binding of primary antibodies was detected using corresponding secondary Alexa Fluor® 488-conjugated mouse anti-rabbit IgG and Alexa Fluor® 647-conjugated donkey anti-goat/rat IgG (Invitrogen). Nuclei were stained with DAPI. The co-localization of HMGB1 and TLR2, or LC3 and lamp-1 were examined using an E2000U confocal microscopy and evaluated using Leica TCS SP2 software [22].

### Statistical Analysis

All values are presented as mean ± SEM. Statistical differences between groups were compared by One Way ANOVA, followed by Student Newman-Keuls post-hoc test. Survival curves were obtained using the Kaplan-Meier method and compared by the log-lank test. A value of p<0.05 was considered significant.

## Results

### Targeting TLR2 and TLR4 Showed Distinct Effects on Acute Dox-induced Mouse Death and Cardiac Dysfunction

We first established an acute mouse model of cardiomyopathy induced by a single injection of Dox (10 mg/kg). At 14 days after Dox injection, the mice displayed remarkable LV dilation and dysfunction, consistent with previous reports [12]. Subsequently, we administered neutralizing antibodies to TLR2, TLR4, or control IgG (Figure 1A). Unexpectedly, administration of the TLR2 and TLR4 Abs to the Dox-treated mice showed opposite results (Figure 1). The mortality rate was significantly lower in the TLR2Ab, but not in the TLR4Ab group, compared with IgG-treated group (Figure 1B, n = 15). Therapeutic blockade of TLR2 displayed attenuated cardiac dysfunction; whereas inhibition of TLR4 aggravated Dox-induced cardiac dysfunction, compared to those treated with IgG (Figure 1C, D). These results indicate that selective blockade of TLR2 activity, but not TLR4, could inhibit Dox-induced cardiac dysfunction, suggesting that TLR2 and TLR4 may be involved in the progression of Dox-induced cardiomyopathy by different mechanisms.

### Targeting TLR2 and TLR4 Differently Regulate Established Cardiac Remodeling in Acute DCM

The mice treated with Dox showed reduced wall thickness and increased LV dimensions (Figure 2A, B), which were significantly attenuated by TLR2Ab treatment. However, TLR4Ab showed opposite effects with TLR2Ab. The heart weight to body weight ratio did not change among the four groups, indicating that the hypertrophic response may not be regulated through TLR signaling pathways (Figure 2C). Dox treatment resulted in a remarkable collagen accumulation and cardiac fibrosis. Blocking TLR2 significantly decreased cardiac fibrosis area, but TLR4Ab exacerbated the fibrotic response (Figure 2D). To evaluate the presence of myofibroblasts, α-SMA immunostaining was performed. As shown in Figure 2E, mice challenged with Dox showed significant up-regulation in the expression of α-SMA in the interstitial non-vessel areas of the myocardium. Administration of the TLR2Ab repressed the expression of α-SMA; whereas blockade of TLR4 increased the expression of α-SMA, compared with IgG-treated groups (Figure 2E). Collectively, our results indicate that targeting TLR2 alleviates, but blocking TLR4 aggravates, the progression of established cardiac remodeling triggered by acute Dox administration.

### Targeting TLR2 Alleviated, but Blocking TLR4 Deteriorated, the Established Chronic Cardiomyopathy

Given the opposite therapeutic efficacy of targeting TLR2 or TLR4 on Dox-induced acute cardiomyopathy, we concentrated on elucidating the possible roles of TLR2 or TLR4 inhibition in Dox-induced chronic cardiomyopathy. We observed that the TLR2Ab administration markedly decreased mortality and attenuated cardiac dysfunction (Figure 3). Conversely, TLR4Ab deteriorated Dox-caused animal death and cardiac malfunction (Figure 3). As shown in Figure 4A and B, cardiomyopathic mice treated with TLR2Ab indicated increased wall thickness and reduced ventricular chamber, compared to IgG-treated mice. However, the heart weight to body weight ratio did not alter following chronic Dox challenge or Ab treatment (Figure 4C). Furthermore, remarkable cardiac interstitial fibrosis occurred in chronic Dox-treated animals was suppressed in TLR2Ab-treated mice (Figure 4D). Similarly, the expression of α-SMA was significantly blunted in TLR2Ab-treated myocardium, compared to IgG-treated group (Figure 4E). However, TLR4Ab treatment induced opposite effects with TLR2Ab on Dox-driven chronic DCM. Taken together, these data further indicate that selective inhibition of TLR2 activity could suppress cardiac remodeling and fibrosis, leading to an amelioration of established chronic cardiomyopathy.

### Targeting TLR2 and TLR4 Differentially Regulated Inflammatory Response

Next, we examined whether inflammation mediated the disparate responses of TLR2 or TLR4 inhibition in Dox-induced cardiomyopathy. Chronic challenge with Dox dramatically induced MCP-1 expression in the myocardium (Figure 5). Administration of TLR2Ab significantly reduced the expression of MCP-1, as compared to IgG-treated samples. To further characterize the immune microenvironment in the hearts, expression levels of IFN-γ, IL-13, TGF-β1 and IL-17A were evaluated. These factors are representative cytokines of the Th1, Th2, Treg, and Th17-type immune responses, respectively [23]. Dox significantly up-regulated the expression of IFN-γ, IL-13, TGF-β1 and IL-17A (Figure 5). TLR2 blockade demonstrated reduced expression of these cytokines. TLR4 blockade showed increased secretion of MCP-1, IL-13 and TGF-β1. These findings indicate that the distinct effects of TLR2 and TLR4 antagonism in Dox-induced chronic cardiomyopathy are associated with differential modulation of inflammation.

### Interruption of TLR2 Signaling Responsible for Therapeutic Efficiency of TLR2 Blocking

TLR2 and TLR4 expression increased after chronic Dox challenge. Functional blockade of TLR2 or TLR4 did not alter the expression levels of TLR2 and TLR4 (Figure 6B). TLR2Ab and TLR4Ab treatment inhibited the downstream signaling, as indicated in Figure 6B. Blocking TLR2, but not TLR4, significantly reduced the expression of endogenous TLR2 and TLR4 agonists HMGB1 and Hsp70 (Figure 6A). Blockade of TLR2 signaling suppressed the interaction of TLR2 with HMGB1, as indicated by co-immunoprecipitation study (Figure 6C) and by confocal microscopy analysis (Figure 6D). These results indicate the TLR2 signaling occurs through HMGB1 and Hsp70. These findings indicate that the interaction of TLR2 and its endogenous agonist liberated from injured cardiac tissue is involved in cardiac remodeling and dysfunction in Dox-induced cardiomyopathy.

### Attenuated Autophagic Signaling Responsible for Aggravated DCM by TLR4 Blockage

To explore if TLR2 and TLR4 blockade regulate Dox-induced DCM by affecting autophagy pathway, we detected the expression and/or activation of the molecules in autophagy signaling. TLR4Ab significantly inhibited cardiac autophagy signaling pathway (Figure 7B). Autophagosome formation was also suppressed in TLR4Ab-treated hearts (Figure 7C). Consistently, TLR4Ab inhibited TRIF-IRF3 signaling pathway and P38 activation, up-regulated activation of mTOR (Figure 7A), which was responsible for a decrease in the autophagic activity in TLR4Ab-treated mice. In contrast, blocking TLR2 had no marked effect on autophagy because TLR2 antagonism resulted in an attenuation of both P38 kinase and PI3K-Akt-mTOR signaling pathway, which relives a feedback regulation of autophagy (Figure 7).

## Discussion

The objective of this study was to evaluate the therapeutic effect of TLR2 and TLR4 blockade on already established Dox induced cardiomyopathy. The most significant findings of our study were the following: (1) blockade of TLR2 attenuated LV dysfunction and fibrosis in Dox triggered acute and chronic cardiomyopathy, which was strongly associated with the reduced inflammation and TLR2 endogenous agonist levels; and (2) In contrast, TLR4 inactivation aggravated Dox-induced cardiac injury and dysfunction, which was related to increased inflammation and decreased autophagy. Combined, our results indicate that TLR2 has detrimental roles in Dox-induced cardiac injury models, while TLR4 has beneficial roles. Therapeutic strategies that block TLR2 but leave TLR4 activity intact, therefore, may provide a novel option to treat heart failure.

Growing studies have documented that Dox induces inflammatory responses in the myocardium [24]. Interestingly, several anti-inflammatory agents such as ketoprofen, dexamethasone and cyclooxygenase-2 inhibitor have protective effects against Dox-triggered myocardial injury, indicating that inflammation plays a key role in Dox induced cardiomyopathy [24], [25]. Activation of TLR4 induces a Th1 type of immune response, which mainly mediates the acute inflammatory response. Stimulation of TLR2, in contrast, induces a mixed Th1, Th2, Treg, and Th17 types of immune response, which mainly mediates the chronic inflammatory and tissue injury responses [26]. In this study, we found that TLR2 antagonism reduced Th1, Th2, Treg and Th17 inflammatory response, which could, at least partly explain the beneficial effect in TLR2Ab treated mice. In contrast, TLR4 inhibition up-regulated Th2 and Treg types inflammation, which contributed to the development and progression of cardiac fibrosis and dysfunction. Therefore, the mechanism underlying different therapeutic effects of targeting TLR2 and TLR4 may be associated with their different inflammatory and immune-regulatory properties. Importantly, we treated after Dox-induced cardiac injury was fulminant, rather than use a pre-treatment strategy. Our experimental design more closely resembles the clinical scenario, where patients would present after symptoms develop. Our results, therefore, have direct translational potential. A previous study showed that TLR4 knockout alleviates Dox induced cardiomyopathy by inhibition of the inflammatory response, which is completely contradictory with this present study [13]. The reasons for contradictory results between two studies are complex as analyzed in our previous study [19]. The possible reason is due to the different timing of TLR4 antagonism between the two studies. Indeed, the preventive (and TLR4 deficiency) or therapeutic inhibition of TLR4 may produce different overcomes in response to TLR4 agonist DOX. The prophylactic antagonism of TLR4 protects against DOX-induced TLR4 activation and TLR4-mediated inflammation and therefore protects from DOX-induced cardiac injury, fibrosis, and dysfunction. However, therapeutic antagonism of TLR4 activity in this model cannot produce an inflammatory-preventing benefit, but it interferes with the TLR4-mediated proresolution of inflammation and cardiac fibrosis [19].

Accumulating clinical studies have indicated that cardiac fibrosis plays a critical role in the pathogenesis of DCM and CHF [27]. The increased deposition of extracellular matrix proteins, such as collagen, within the myocardium led to an increased stiffening of the LV that induces both systolic and diastolic dysfunction [28]. TGF-β1 is involved in LV remodeling by facilitating collagen synthesis and inhibiting collagen degradation [29]. Blocking IL-17A facilitates the resolution of pulmonary inflammation and fibrosis [30]. In this study, blocking TLR2 showed reduced levels of both TGF-β1 and IL-17A, which is consistent with reduced cardiac fibrosis; but blocking TLR4 increased TGF-β1 levels, which promotes collagen accumulation in the myocardium.

Recent studies indicate that damage-associated molecular pattern molecules (DAMPs) are proteins released from an injured tissue to participate in the pathogenesis of multiple diseases through the activation of TLRs [31]. HMGB1 is a DAMP molecule that participates in the pathogenesis of arthritis and atherosclerosis by regulating immune response [32], [33]. HMGB1 serves as an endogenous ligand of both TLR2 and TLR4, and mediates the response to infection, injury and inflammation [34]. In this study, we demonstrated that blocking TLR2, but not TLR4 significantly suppressed the expression of HMGB1, suggesting a positive feedback loop when TLR2 is the receptor the HMGB1 binds. Furthermore, TLR2Ab inhibited the interaction between TLR2 and HMGB1, which blocked the DAMPs signaling pathway. These findings suggest that HMGB1 may implicate in Dox induced cardiomyopathy by activating TLR2 to amplify the inflammatory response. However, we cannot exclude the possibility that other DAMPs, such as Hsp60 or Hsp70, are also implicated in Dox induced DCM by stimulating TLR2.

Autophagy provides an alternative mechanism for cell survival in cardiac remodeling [35], [36]. Interestingly, the detrimental effect of targeting TLR4 was associated with a suppression of autophagy, indicating that autophagy activation mediated by TLR4 signaling plays an important role in Dox evoked DCM. Our findings showed that TLR4-mediated basal autophagic activity is critically required for resolving chronic inflammation and fibrosis from injured tissues [19]. TLR4-activated autophagy is mediated by MyD88-p38 kinase-dependent and TRIF-dependent pathways [37]. Indeed, we found that TLR4Ab blocked not only the MyD88-p38 kinase pathway, but also the TLR4-TRIF-IRF3 pathway, which caused a suppression of autophagy in cardiac myocytes (Figure 8). In contrast, two distinct downstream pathways, MyD88-NF-κB and PI3K-Akt-mTOR, mediated TLR2 signaling. Activation of TLR2 induced autophagy through activation of P38 kinase, but inhibited autophagy by activating the PI3K-Akt-mTOR pathway [38]. Hence, blocking TLR2 causes a biphasic regulation on autophagic activity because of inhibition of both P38 kinase and PI3K-Akt-mTOR activities (Figure 8). Our work indicates that differentially regulating autophagy may be responsible for the opposite effects observed by TLR2 and TLR4 antagonism on established Dox-DCM.

A recent study has reported that intraperitoneal injection of DOX causes severe small intestinal mucositis, which is attenuated by TLR2 and TLR9 deficiency [39]. This observation reminds that more caution should be given when one uses this model and particularly interprets the experimental data and derive the conclusion from this model. Because the treatment of these animals was initiated at the two weeks after DOX injection even in acute DCM study, the DOX-induced acute inflammation in the injection site should be resolved or diminished. Thus, although abdominal injection of DOX may cause abdomen inflammation, it does not interfere with the DCM development. Whatever, using animal models mimicking human cardiomyopathy should be better for the translation of the basic research findings to clinical patients.

### Conclusions

In summary, our study provides direct evidence of the differential effects of TLR2 or TLR4 inhibition on Dox-induced acute and chronic cardiomyopathy. In particular, TLR4 activity was crucial for the resolution of inflammation and cardiac fibrosis, while blocking TLR2 inhibited specific immune responses induced by Dox to attenuate already-established ventricular remodeling and dysfunction. Our findings indicate that TLR2 may be a promising therapeutic target, while TLR4 is an anti-target, for the development of agents against cardiac remodeling in DCM and CHF.
