# Correction: Toll-Like Receptor (TLR) 2 and TLR4 Differentially Regulate Doxorubicin Induced Cardiomyopathy in Mice

**DOI:** 10.1371/annotation/e82f77a8-3d29-44be-a9ef-7abc6c7e584a

**Published:** 2012-10-11

**Authors:** Yonggang Ma, Xiaowei Zhang, Huayan Bao, Su Mi, Wenfeng Cai, Huimin Yan, Qingqing Wang, Ziyan Wang, Jun Yan, Guochang Fan, Merry L. Lindsey, Zhuowei Hu

There was an error in the tenth author's name. Guo-Chang Fan is correct.

